# Next-Generation Immunotherapy: Advancing Clinical Applications in Cancer Treatment

**DOI:** 10.3390/jcm13216537

**Published:** 2024-10-30

**Authors:** Pankaj Garg, Siddhika Pareek, Prakash Kulkarni, David Horne, Ravi Salgia, Sharad S. Singhal

**Affiliations:** 1 Department of Chemistry, GLA University, Mathura 281406, Uttar Pradesh, India; 2 Departments of Medical Oncology & Therapeutics Research, Beckman Research Institute of City of Hope, Comprehensive Cancer Center and National Medical Center, Duarte, CA 91010, USA; 3 Departments of Molecular Medicine, Beckman Research Institute of City of Hope, Comprehensive Cancer Center and National Medical Center, Duarte, CA 91010, USA

**Keywords:** next-generation immunotherapy, immune checkpoint inhibitors, CAR-T cell therapy, cancer vaccines, immune modulation

## Abstract

Next-generation immunotherapies have revolutionized cancer treatment, offering hope for patients with hard-to-treat tumors. This review focuses on the clinical applications and advancements of key immune-based therapies, including immune checkpoint inhibitors, CAR-T cell therapy, and new cancer vaccines designed to harness the immune system to combat malignancies. A prime example is the success of pembrolizumab in the treatment of advanced melanoma, underscoring the transformative impact of these therapies. Combination treatments, integrating immunotherapy with chemotherapy, radiation, and targeted therapies, are demonstrating synergistic benefits and improving patient outcomes. This review also explores the evolving role of personalized immunotherapy, guided by biomarkers, genomic data, and the tumor environment, to better target individual tumors. Although significant progress has been made, challenges such as resistance, side effects, and high treatment costs persist. Technological innovations, including nanotechnology and artificial intelligence, are explored as future enablers of these therapies. The review evaluates key clinical trials, breakthroughs, and the emerging immune-modulating agents and advanced delivery systems that hold great promise for enhancing treatment efficacy, reducing toxicity, and expanding access to immunotherapy. In conclusion, this review highlights the ongoing advancements in immunotherapy that are reshaping cancer care, with future strategies poised to overcome current challenges and further extend therapeutic reach.

## 1. Introduction

Immunotherapy has transformed cancer treatment by harnessing the body’s immune system to identify and destroy cancer cells. Unlike conventional methods such as chemotherapy and radiation, which indiscriminately target both healthy and cancerous cells, immunotherapy provides a more targeted and often more efficacious approach [1]. The method works by either boosting or modulating the immune system, enabling it to specifically target cancer cells while sparing surrounding healthy tissues [2]. Among the most prominent forms of immunotherapy are immune checkpoint inhibitors (ICIs), which block proteins (such as PD-1, PD-L1, and CTLA-4) that cancer cells or immune cells use to weaken the immune response, allowing a more robust attack on tumors. CAR-T cell therapy entails modifying a patient’s T cells at the genetic level to express chimeric antigen receptors, boosting their capacity to detect and destroy cancer cells. Cancer vaccines aim to activate the immune system to recognize cancer-associated antigens, triggering an immune response against tumor cells. However, earlier vaccines faced challenges such as low immunogenicity and limited effectiveness in solid tumors, which limited their application. Today, improvements in understanding tumor antigens and adjuvants are helping to overcome these limitations, reinvigorating interest in cancer vaccines. Monoclonal antibodies are lab-created proteins that bind to specific markers on cancer cells, signaling the immune system to target them for elimination [3].

Immunotherapy has revolutionized the treatment landscape for a range of cancers, especially melanoma, non-small cell lung cancer (NSCLC), and renal cell carcinoma, as well as hematologic malignancies such as leukemia and lymphoma. In these cancers, ICIs like nivolumab and pembrolizumab have dramatically extended survival rates where traditional therapies had failed, with some patients achieving long-term remission. For instance, in metastatic melanoma, five-year survival rates have significantly improved due to therapies targeting PD-1, while in NSCLC, ICIs have shown survival benefits, even in advanced stages of the disease [4]. Since immunotherapy enhances the immune system’s ability to fight cancer rather than directly targeting cells, it generally results in fewer side effects, providing patients with an improved quality of life during treatment [5]. The field of tumor immunotherapy has evolved significantly, moving from early, modest approaches like cancer vaccines and cytokine treatments to major breakthroughs in ICIs. Therapies targeting PD-1 and CTLA-4 have drastically improved the outcomes in cancers like melanoma and lung cancer, while the advent of CAR-T cell therapy has demonstrated exceptional success in treating blood cancers, particularly in relapsed or refractory acute lymphoblastic leukemia and large B-cell lymphoma [6]. However, early immunotherapies were limited by issues such as poor response rates, high relapse rates, and significant toxicity. These limitations often stemmed from the inability to sustain durable immune responses, particularly in solid tumors.

Next-generation immunotherapy represents a pivotal advancement in improving cancer treatment outcomes, addressing challenges such as resistance, side effects, and limited applicability. These cutting-edge therapies counteract resistance by targeting tumor heterogeneity and activating multiple immune pathways, enhancing response rates. Resistance to earlier therapies was often due to tumor adaptation to evade immune detection or the creation of an immunosuppressive microenvironment. Current strategies focus on overcoming these obstacles by targeting immune-suppressive cells within the tumor microenvironment (TME) and reprogramming immune cells to sustain a prolonged attack. They also focus on minimizing immune-related side effects by utilizing selective targeting and immune modulation to reduce toxicity [7]. Precision has been further refined with biomarker-driven, personalized treatments, enabling therapies to be tailored to the specific characteristics of a patient’s tumor. Despite these advancements, managing immune-related adverse events (irAEs), which can range from mild to life-threatening, remains a significant challenge that researchers are actively working to mitigate [8]. The growing trend of combination therapies, like pairing checkpoint inhibitors with CAR-T cells, shows promise for treating cancers that have been historically difficult to manage. Additionally, emerging technologies such as nanotechnology and artificial intelligence (AI) are improving drug delivery systems and accelerating the creation of more effective treatments [9]. Significantly, next-generation immunotherapy is broadening its scope to include previously resistant diseases like pancreatic and ovarian cancers, solidifying its role in modern cancer care. Its ability to modulate the immune system through the selective targeting of suppressive immune cells within the TME offers new possibilities for treating these cancers [10].

## 2. Mechanisms of Tumor Immunotherapy (Immune Response in Cancer)

### 2.1. Immune Surveillance in Cancer

The immune system is integral in maintaining bodily homeostasis, constantly surveilling for abnormal or malignant cells through a process known as immune surveillance. In a healthy immune response, cytotoxic T lymphocytes (CTLs) and natural killer (NK) cells detect and destroy emerging cancer cells before they proliferate into full-blown tumors [11]. The activation of these immune cells relies on the recognition of tumor-associated antigens applied to cancer cells, triggering a cascade of immune responses aimed at their elimination. This surveillance system is often successful in identifying and neutralizing nascent tumors, helping to maintain immune equilibrium.

### 2.2. Tumor Immune Evasion Mechanisms

Despite the immune system’s constant surveillance, cancer cells have evolved complex strategies to evade immune detection and destruction, a process referred to as immune escape. Tumors utilize various mechanisms to suppress or evade the immune system, allowing them to grow unchecked. One key strategy involves the creation of an immunosuppressive tumor microenvironment (TME), which impairs the immune system’s ability to recognize and eliminate cancer cells. Within the TME, immune-suppressive cells such as regulatory T cells (Tregs), myeloid-derived suppressor cells (MDSCs), and immunosuppressive cytokines contribute to tumor progression by dampening the body’s natural immune defenses [12]. Tumors also exploit immune checkpoint pathways—natural mechanisms designed to prevent the over activation of the immune system and autoimmunity. Proteins such as PD-1 and CTLA-4 act as brakes on T cell activity, reducing immune responses when engaged. Tumor cells evade immune detection by expressing ligands like PD-L1, which binds to PD-1 receptors on T cells, effectively downregulating their activity and promoting tumor immune tolerance [13]. The PD-1/PD-L1 interaction, in particular, is a critical pathway that tumors exploit to suppress T cell function and prevent tumor destruction. By binding PD-L1 to the PD-1 receptor on the T cells, tumors inhibit T cell proliferation and cytokine production, thereby creating an immune-resistant environment. Additionally, the CTLA-4 checkpoint pathway functions by limiting the priming of T cells by antigen-presenting cells, further weakening the immune response. This multifaceted immune evasion requires innovative therapeutic strategies to counteract its effects.

### 2.3. Therapeutic Strategies in Tumor Immunotherapy

Immunotherapy has emerged as a revolutionary approach to overcoming cancer’s defenses by harnessing and boosting the immune system’s natural ability to fight the disease. The primary goal of tumor immunotherapy is to reawaken immune cells, particularly T cells, enabling them to recognize and effectively eliminate cancer cells [14]. Immune checkpoint inhibitors (ICIs), such as pembrolizumab and nivolumab, have been pivotal in this effort. By blocking the PD-1/PD-L1 and CTLA-4 pathways, these drugs lift the immune “brakes,” allowing T cells to mount a more robust attack against cancer cells. The success of ICIs has been particularly remarkable in melanoma and NSCLC, where traditional therapies have often fallen short. Clinical trials such as CheckMate 057 and KEYNOTE-001 have demonstrated significantly improved survival rates in patients treated with ICIs, establishing these therapies as a cornerstone in modern cancer care [15]. Another significant advancement in immunotherapy is chimeric antigen receptor T cell (CAR-T) therapy, where a patient’s T cells are genetically engineered to express CARs that enable them to specifically target and destroy cancer cells [16,17]. This approach has achieved unprecedented success in treating hematologic cancers such as acute lymphoblastic leukemia (ALL) and large B-cell lymphoma, with the ZUMA-1 trial demonstrating remarkable outcomes in refractory large B-cell lymphoma [18]. Researchers are now exploring ways to adapt CAR-T cell therapy for use in solid tumors, which pose greater challenges due to the immunosuppressive TME and tumor heterogeneity.

### 2.4. Addressing Tumor Heterogeneity and Resistance

The success of immunotherapy is not only dependent on activating the immune system but also on overcoming the inherent complexity of cancer biology. Tumor heterogeneity, or the existence of diverse cell populations within a single tumor, presents a significant challenge for immunotherapy. Different subclones within a tumor may express varying levels of immune evasion markers, respond differently to therapy, or even develop resistance to immunotherapeutic approaches [19]. For example, some tumor cells may lack the PD-L1 ligand, rendering them less susceptible to PD-1 inhibitors. To address this, personalized immunotherapy has gained prominence, leveraging biomarkers and genomic profiling to tailor treatments to the specific characteristics of a patient’s tumor. Precision immunotherapy has shown promise in reducing resistance and improving long-term survival outcomes by targeting specific mutations or pathways that drive immune evasion in individual tumors [20]. Moreover, combination therapies that pair ICIs with chemotherapy, radiation, or targeted agents have shown synergistic effects, enhancing treatment efficacy. For example, combining pembrolizumab with chemotherapy in the KEYNOTE-189 trial demonstrated superior survival outcomes in NSCLC [21].

### 2.5. Challenges and Future Directions

Despite its promise, immunotherapy faces challenges, including primary and acquired resistance. Primary resistance refers to patients who do not respond to immunotherapy from the outset, while acquired resistance develops after an initial positive response to treatment. Research is ongoing to understand the mechanisms behind these forms of resistance, which may involve the loss of antigen presentation, the upregulation of alternative immune checkpoints, or changes in the TME. To overcome resistance, integrating immunotherapy with other treatment modalities such as targeted therapy, radiation, and chemotherapy is becoming a standard approach for many cancers [22]. Future directions include the development of next-generation ICIs and engineered T cell therapies that can target multiple immune evasion pathways simultaneously, as well as efforts to apply immunotherapy to traditionally resistant cancers like pancreatic and ovarian cancer.

In summary, the mechanisms behind tumor immunotherapy are closely tied to the immune system’s natural ability to detect and eliminate cancer cells. By countering the immune evasion strategies used by cancer cells, immunotherapy reactivates and strengthens the immune response, offering a more precise and durable form of cancer treatment. As advances continue, especially in areas like ICIs, CAR-T cell therapy, and personalized immunotherapy, the future of cancer care looks increasingly optimistic, with the potential for more targeted and effective treatments that not only attack tumors but also provide long-term protection against their return (Table 1).

## 3. Emerging Next-Generation Immunotherapy Approaches

**Figure 1:**
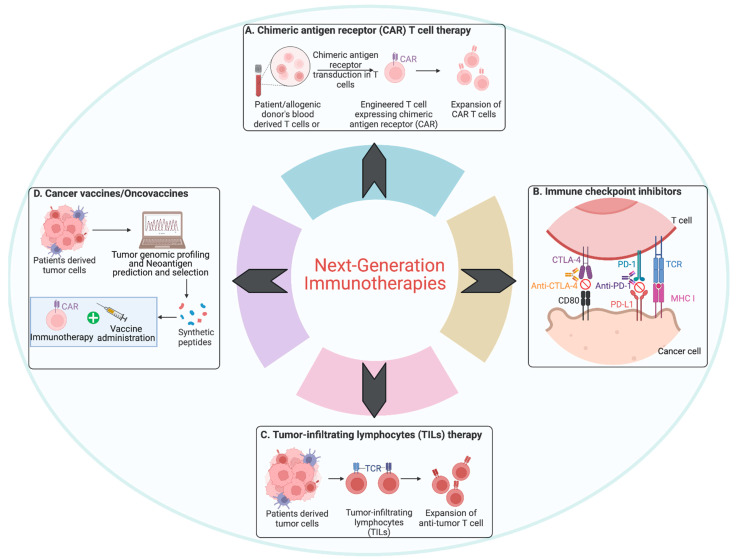
**Overview of next-generation immunotherapy strategies.** (**A**) Chimeric antigen receptor (CAR) T cell therapy: Patient or donor-derived T cells are engineered to express CARs that recognize tumor antigens, followed by expansion of these modified T cells for re-administration. (**B**) Immune checkpoint inhibitors (ICIs): Tumor cells display PD-L1 and CD80 as immune surveillance evasion strategies. They interact with PD-1 and CTLA-4 displayed on T cells which serve as immune checkpoints that inhibit immune responses. ICIs such as anti-CTLA-4 and anti-PD-1/PD-L1 block receptors on T cells, preventing tumor-induced immune suppression and restoring T cell-mediated cytotoxicity. (**C**) Tumor-infiltrating lymphocytes (TILs) therapy: TILs isolated from patient tumors are expanded ex vivo before being reinfused into the patient to enhance anti-tumor activity. (**D**) Cancer vaccines/oncovaccines: Personalized cancer vaccines, derived from tumor cells, are sequenced, and neoantigens are identified to produce synthetic peptides, formulated into personalized vaccines capable of generating an immune response against tumor-specific antigens. Vaccines can be used in conjunction with CAR-T cells or other immunotherapies for enhanced efficacy.

The field of immunotherapy is undergoing rapid advancements, with novel approaches aimed at improving the efficacy and range of treatments available for various cancer types. Prominent strategies include CAR-T cell therapy, immune checkpoint inhibitors (ICIs), tumor-infiltrating lymphocytes (TIL) therapy, and cancer vaccines. These methods mark significant progress in leveraging the immune system to fight cancer more effectively [31] (Figure 1).

### 3.1. CAR-T Cell Therapy: Recent Advances and Applications

CAR-T (chimeric antigen receptor T cell) therapy is a groundbreaking immunotherapy that involves modifying a patient’s T cells to express receptors specifically targeting cancer cells. These engineered T cells are then reinfused into the patient to seek and destroy malignant cells. Originally used for blood cancers like leukemia and lymphoma, CAR-T therapies such as Yescarta and Kymriah have shown remarkable success, especially in cases resistant to other treatments [23]. However, there are ongoing challenges with managing tumor relapse, reducing off-tumor toxicities, and expanding the therapy’s efficacy for solid tumors, which remain difficult to treat [32]. Recent advancements focus on improving both the precision and safety of CAR-T cells. One key innovation is the development of bispecific CAR-T cells which are engineered to target two antigens simultaneously. This dual-targeting mechanism reduces the chances of tumor escape, where cancer cells lose the antigen targeted by traditional CAR-T cells to avoid detection. Another advancement involves CARs equipped with “on-switches” and “off-switches” that provide dynamic control over T cell activation. The “on-switch” ensures that the T cells are active only when near cancer cells, while the “off-switch” can halt their activity to prevent adverse side effects, such as cytokine release syndrome (CRS). These modifications significantly enhance both the safety and therapeutic efficacy of CAR-T therapies, and clinical trials are currently underway to test the next generation of CAR-T cells, particularly in solid tumors [33].

### 3.2. Immune Checkpoint Inhibitors (ICIs): Expanding Beyond PD-1 and CTLA-4

ICIs have transformed cancer treatment by targeting proteins like PD-1 and CTLA-4, which cancer cells exploit to suppress the immune response. ICIs have shown success in treating cancers like melanoma, renal cell carcinoma, and NSCLC by preventing these proteins from inhibiting T cells [34]. However, newer research has expanded the focus beyond PD-1 and CTLA-4 to other checkpoint pathways such as TIM-3, LAG-3, and TIGIT [35,36]. Blocking these alternative checkpoints offers hope for patients who do not respond to traditional ICIs. For instance, LAG-3 inhibitors, such as relatlimab, have shown promise in enhancing T cell function and are being evaluated in several ongoing clinical trials, including studies on melanoma and ovarian cancer [37]. These trials aim to determine whether combining inhibitors of multiple checkpoints can overcome resistance mechanisms and provide a more robust and sustained immune response in various cancer types (Table 2). Additionally, targeting multiple checkpoints simultaneously is being explored in combination therapies, which may unlock treatments for more resistant cancers, such as colorectal and pancreatic cancers [38].

### 3.3. Tumor-Infiltrating Lymphocyte (TIL) Therapy: Challenges and Advances

TIL therapy involves harvesting a patient’s own immune cells that have naturally infiltrated the tumor and expanding them ex vivo before reinfusing them to enhance their anti-tumor capabilities. TIL therapy has demonstrated significant potential in treating metastatic melanoma, providing long-term remissions, even in patients resistant to other therapies [50]. However, extending the success of TIL therapy to solid tumors remains a significant challenge due to the immunosuppressive nature of the tumor microenvironment (TME) in these cancers. Solid tumors often create physical and chemical barriers that hinder the effectiveness of TILs, including dense stromal tissue and the presence of immunosuppressive cytokines and cells such as myeloid-derived suppressor cells (MDSCs) and Tregs. These factors contribute to a hostile environment that inhibits the function of TILs once reinfused [51]. Researchers are actively investigating strategies to overcome these barriers, such as combining TIL therapy with ICIs to enhance the function of the reinfused T cells and improve their ability to infiltrate and destroy solid tumors. Ongoing clinical trials are also evaluating the application of TIL therapy in other cancers, including cervical and head and neck cancers, with early results showing promise [24].

### 3.4. Cancer Vaccines/Oncovaccines: From Prophylactic to Therapeutic Uses

Cancer vaccines, traditionally used to prevent virus-induced cancers (e.g., HPV vaccines), are now being developed as therapeutic vaccines to target existing cancers. These vaccines aim to stimulate the immune system by presenting tumor-specific antigens, enhancing the body’s ability to detect and attack cancer cells [24]. Therapeutic cancer vaccines, such as sipuleucel-T, have shown efficacy in treating prostate cancer, while experimental vaccines are being explored for lung, breast, and pancreatic cancers [25]. One of the main challenges in developing effective cancer vaccines is the ability of cancer cells to mutate and evade immune detection. Personalized cancer vaccines, which target neoantigens—tumor-specific mutations unique to each patient—are emerging as a promising solution. These personalized vaccines are designed using genomic sequencing to tailor the immune response to an individual’s tumor profile, improving the precision and efficacy of the treatment. Researchers are also exploring combinations of cancer vaccines with ICIs or CAR-T therapy to amplify the immune response, potentially improving clinical outcomes and offering long-term protection against cancer recurrence [52].

In summary, emerging immunotherapies such as CAR-T cell therapy, ICIs, TIL therapy, and cancer vaccines are pushing the boundaries of cancer treatment. By leveraging advanced technologies and combination strategies, these therapies have the potential to improve patient outcomes significantly (Figure 1). Ongoing research and clinical trials continue to refine these approaches, offering hope for more personalized and effective cancer treatments.

## 4. Combination Therapies in Cancer Treatment

**Figure 2:**
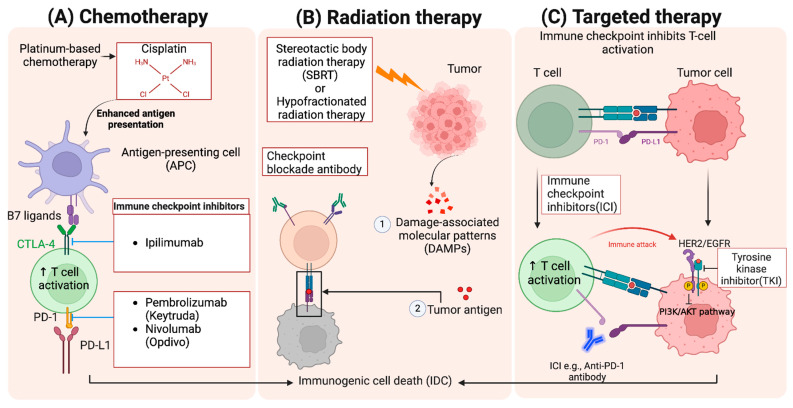
**Combination therapies in cancer treatment.** This figure illustrates the integration of chemotherapy, radiation therapy, and targeted therapy with immunotherapy to enhance anti-tumor response. (**A**) Platinum-based chemotherapeutics such as cisplatin increase antigen presentation via antigen-presenting cells (APCs), leading to enhanced T cell activation when combined with CTLA-4 and PD-1 inhibitors like ipilimumab, pembrolizumab, and nivolumab. (**B**) Radiation therapy promotes immunogenic cell death (IDC) through damage-associated molecular patterns (DAMPs) and tumor antigen (TAs) release, boosting the immune response. (**C**) Combining immune checkpoint inhibitors (ICIs) targeting the PD-1/PD-L1 and targeted drugs, such as tyrosine kinase inhibitors (TKIs) targeting HER2/EGFR mutations, further enhances T cell-mediated immune attack through inhibition of the PI3K/AKT pathway.

Combining immunotherapy with other cancer treatments has emerged as a powerful strategy to enhance the effectiveness of treatments and overcome the limitations of monotherapies. These combination approaches aim to create synergistic effects that improve response rates, reduce resistance, and provide longer-lasting benefits to patients. The following are three key combinations: immunotherapy with chemotherapy, immunotherapy with radiation, and the integration of immunotherapy with targeted therapies [28] (Figure 2).

### 4.1. Immunotherapy with Chemotherapy

The combination of immunotherapy with chemotherapy has shown significant promise in improving cancer treatment outcomes. Chemotherapy, traditionally known for its role in directly killing rapidly dividing cancer cells, has long been a cornerstone of cancer treatment. Nevertheless, chemotherapy may impair the immune system, which can hinder the body’s ability to generate a strong immune response against cancer. Despite this drawback, recent research has shown that chemotherapy-induced immunogenic cell death (ICD) can prime the immune system to be more receptive to immunotherapy. ICD occurs when dying cancer cells release a range of signals, such as tumor antigens, DAMPs (damage-associated molecular patterns), and other immune-activating molecules. These signals stimulate dendritic cells and other antigen-presenting cells, which, in turn, activate cytotoxic T cells to recognize and destroy cancer cells more effectively. This priming effect enhances the subsequent response to immune checkpoint inhibitors (ICIs), CAR-T therapies, and other immunotherapeutic agents [26].

Chemotherapy can also diminish the presence of Tregs and MDSCs, which typically inhibit immune responses in the TME. This reduction in immune suppression enables immunotherapy agents to work more effectively [27]. Specific clinical examples of this combination have shown success across multiple cancers. One of the best-known examples is the use of ICIs like pembrolizumab (Keytruda) or nivolumab (Opdivo) alongside platinum-based chemotherapy. In NSCLC, this combination has led to significant improvements in overall survival (OS) and progression-free survival (PFS). For example, in the KEYNOTE-189 trial, pembrolizumab combined with chemotherapy resulted in a 51% reduction in the risk of death compared to chemotherapy alone, with a median OS of 22 months vs. 10.7 months. Similar success has been observed in bladder cancer, triple-negative breast cancer (TNBC), and gastric cancer, where chemotherapy primes the tumor for a stronger immune response. In the IMpassion130 trial, the combination of atezolizumab with nab-paclitaxel demonstrated a 7.5-month improvement in OS for patients with metastatic TNBC expressing PD-L1. Ongoing research is exploring how to optimize the timing, dosing, and sequencing of chemotherapy and immunotherapy to minimize side effects and enhance their synergistic potential. The understanding of how these two modalities work together continues to open new avenues for treating a broader range of cancers [53].

### 4.2. Immunotherapy and Radiation: Synergistic Effects

The combination of immunotherapy and radiation therapy represents another promising approach to cancer treatment. Radiation has been a standard treatment for many types of cancer, particularly for localized tumors. Traditionally, radiation works by damaging the DNA of cancer cells, leading to cell death. Recent studies have demonstrated that radiation can also have profound immunomodulatory effects. By inducing immunogenic cell death (ICD), radiation therapy releases tumor-associated antigens (TAAs) and DAMPs, which enhance the immune system’s ability to detect and target cancer cells. This process helps to “convert” the tumor into an in situ vaccine that primes an immune response. Moreover, radiation can stimulate the expression of interferon and other cytokines, which increase the infiltration of cytotoxic T cells into the TME, making the tumor more susceptible to attack by immunotherapeutic agents [54]. One of the most notable synergies between immunotherapy and radiation is seen in the use of ICIs in combination with stereotactic body radiation therapy (SBRT) or hypofractionated radiation therapy. For instance, in melanoma, the combination of anti-PD-1 therapy with radiation has shown improved response rates and prolonged survival in patients who previously did not respond to immunotherapy alone [55,56]. In studies of NSCLC, combining PD-1 or PD-L1 inhibitors (such as pembrolizumab or durvalumab) with radiation has resulted in significant local tumor control and systemic immune responses. The PACIFIC trial demonstrated that the addition of durvalumab after chemo-radiotherapy in stage III NSCLC improved 5-year OS rates by 42.9%, compared to 33.4% for chemo-radiotherapy alone [57]. Similarly, in head and neck cancers, this combination has led to better outcomes by enhancing local and systemic tumor regression through the abscopal effect. The abscopal effect occurs when localized radiation treatment, driven by systemic immune activation, leads to the regression of not only the irradiated tumor but also distant metastases [58]. The future of this combination therapy lies in identifying the best doses, fractions, and timing for radiation and immunotherapy to maximize patient outcomes while minimizing toxicity.

### 4.3. Targeted Therapy and Immunotherapy: New Horizons

The combination of targeted therapy and immunotherapy is at the forefront of modern cancer treatment. Targeted therapies work by specifically blocking the molecular pathways that drive cancer growth and survival, unlike chemotherapy or radiation, which affect both cancerous and healthy cells. However, targeted therapies can also play a role in altering the immunosuppressive TME, making it more favorable for immune responses. For example, inhibitors of the PI3K/AKT/mTOR pathway can decrease the recruitment of MDSCs and Tregs, which are key drivers of immune evasion in many cancers [29]. Similarly, tyrosine kinase inhibitors (TKIs) targeting the EGFR pathway can help modulate immune cells and enhance the infiltration of effector T cells into tumors [59].

In cancers driven by mutations in the PI3K/AKT/mTOR pathway or MAPK pathway, targeted therapies help suppress these oncogenic signals. However, these mutations often lead to an immunosuppressive TME. By combining targeted therapies with ICIs, researchers aim to reverse immune suppression and enhance T cell activity within the tumor. This combination has shown potential in treating melanoma, renal cell carcinoma, and colorectal cancer [30]. One significant advantage of targeted therapy and immunotherapy combinations is the ability to address tumor heterogeneity—the existence of genetically diverse cancer cells within the same tumor [60]. For example, in the COMBI-i trial, the combination of dabrafenib and trametinib with the PD-1 inhibitor spartalizumab demonstrated a 67% overall response rate (ORR) in patients with BRAF-mutant melanoma. Targeted therapies can reduce the prevalence of certain tumor subclones, while immunotherapy can address the remaining subclones, creating a more comprehensive treatment approach. Moreover, these combinations are being explored in precision medicine frameworks, in which the genetic profiling of tumors allows for more tailored treatments, based on specific mutations or biomarkers. The use of genetic profiling to identify actionable mutations, combined with immunotherapy, has been particularly promising in cancers such as NSCLC and colorectal cancer [61]. As research in this area progresses, many clinical trials are underway to identify the most effective combinations and treatment regimens. Dual inhibition strategies, where multiple molecular pathways are targeted alongside immune modulation, represent a promising area for future cancer therapies [62].

## 5. Clinical Trials and Advances Supporting Next-Generation Immunotherapies

Advances in next-generation immunotherapies, such as CAR-T cell therapy and ICIs, represent a paradigm shift in cancer treatment. These innovative therapies harness the immune system’s capacity to recognize and destroy cancer cells, offering new hope, particularly for patients with refractory or advanced disease [63]. The evolving clinical landscape highlights not only the remarkable efficacy of these treatments but also the challenges associated with managing their toxicity and integrating them into real-world clinical practice. Below, the key trials and their impact on both clinical outcomes and practical implementation is examined.

### 5.1. CAR-T Cell Therapy Trials: High Efficacy with Complex Toxicity Management

CAR-T (chimeric antigen receptor T cell) therapy has emerged as a breakthrough in the treatment of hematologic malignancies, particularly in relapsed or refractory large B-cell lymphoma [64]. Early landmark clinical trials, such as ZUMA-1, JULIET, and TRANSCEND, have demonstrated unprecedented efficacy in these patients, who previously had very limited treatment options [65,66].

ZUMA-1 trial: In this pivotal study evaluating axicabtagene ciloleucel (axi-cel) in patients with relapsed/refractory large B-cell lymphoma, an overall response rate (ORR) of 83% was observed, with a complete response (CR) rate of 58%. At 2 years of follow-up, 40% of patients remained in remission [43,44,67]. 

JULIET trial: This trial, assessing the efficacy of tisagenlecleucel, reported similar success, with an ORR of 52% and a CR rate of 40% in a heavily pretreated patient population [45,46].

TRANSCEND trial: Liso-cel, a defined composition CAR-T product, demonstrated a CR rate of approximately 54%, with manageable toxicity, positioning it as a promising option in the treatment landscape [68].

Despite these impressive results, CAR-T therapies are associated with significant toxicities, particularly cytokine release syndrome (CRS) and immune effector cell-associated neurotoxicity syndrome (ICANS). CRS occurs in the majority of patients, with varying severity. Mild cases present with fever and fatigue, while more severe cases can lead to life-threatening hypotension, hypoxia, and multi-organ dysfunction. Neurotoxicity, ranging from confusion to seizures and coma, also remains a considerable concern. Toxicity management has become a central focus in the clinical application of CAR-T therapy. CRS is now commonly managed with the anti-IL-6 receptor monoclonal antibody tocilizumab, which has been approved for CRS management based on its ability to reverse symptoms rapidly. Corticosteroids are also employed, particularly in cases of severe CRS or neurotoxicity. The development of standardized management protocols has been crucial in reducing CAR-T therapy-related morbidity and improving patient outcomes.

### 5.2. Immune Checkpoint Inhibitors (ICIs): Key Trials and Broader Impact

Immune checkpoint inhibitors, which target inhibitory pathways such as PD-1/PD-L1 and CTLA-4, have revolutionized the treatment of several solid tumors, including NSCLC, melanoma, renal cell carcinoma, and more. The CheckMate and KEYNOTE trials are cornerstone studies that have demonstrated the broad efficacy of ICIs, reshaping treatment paradigms across multiple cancer types.

CheckMate-067 trial: This trial assessed the combination of nivolumab (anti-PD-1) and ipilimumab (anti-CTLA-4) in patients with advanced melanoma. At 5 years of follow-up, the combination therapy resulted in an overall survival (OS) rate of 52%, compared to 44% for nivolumab alone and 26% for ipilimumab alone. This trial established combination immunotherapy as a highly effective approach in treating advanced melanoma, albeit with a higher incidence of immune-related adverse events (irAEs) [69,70].

KEYNOTE-189 trial: In metastatic NSCLC, the combination of pembrolizumab (anti-PD-1) with chemotherapy demonstrated a significant survival benefit. At 22 months of median follow-up, the OS was 22 months for the pembrolizumab plus chemotherapy group, compared to 10.7 months for chemotherapy alone [71]. Importantly, the addition of pembrolizumab did not result in a marked increase in severe toxicity, making it a viable frontline treatment option for metastatic NSCLC patients with PD-L1 expression ≥1% [72,73]. 

CheckMate-025 trial: In advanced renal cell carcinoma, nivolumab outperformed everolimus, a standard second-line therapy, with a median OS of 25 months compared to 19.6 months, respectively, while maintaining a favorable toxicity profile. This led to the widespread adoption of nivolumab in renal cell carcinoma treatment [74].

Toxicity management with ICIs revolves around the control of immune-related adverse events (irAEs), which can affect any organ system. Common irAEs include dermatitis, colitis, pneumonitis, and hepatitis. These toxicities are generally managed with immunosuppressive therapies such as corticosteroids, with the goal of preventing irreversible damage while maintaining anti-tumor efficacy. For severe or refractory irAEs, additional immunosuppressants like infliximab or mycophenolate may be used [75].

### 5.3. Cancer Vaccine Trials

The sipuleucel-T (Provenge) vaccine represents a significant advancement in the treatment of metastatic castration-resistant prostate cancer (mCRPC) and was evaluated in the pivotal IMPACT (Immunotherapy for Prostate Adenocarcinoma Treatment) trial [76]. This vaccine works by boosting the patient’s immune system, using the patient’s own immune cells, which are modified with a recombinant fusion protein comprising prostatic acid phosphatase (PAP)—a common prostate cancer antigen—and granulocyte-macrophage colony-stimulating factor (GM-CSF) [77]. In the IMPACT trial, sipuleucel-T demonstrated a median survival extension of 4.1 months compared to that of the placebo group (25.8 months vs. 21.7 months), with a 22% reduction in the risk of death [78]. While sipuleucel-T is generally well-tolerated, with common side effects including chills, fever, and headaches during infusion, it does not cause significant tumor shrinkage or halt disease progression. However, its ability to extend survival marked a breakthrough in prostate cancer treatment, leading to its FDA approval in 2010 as the first therapeutic cancer vaccine [79].

Despite these successes, its broader adoption has been challenged by the high production costs and the complex, labor-intensive process of personalizing each dose. Nevertheless, the IMPACT trial demonstrated the potential of cancer vaccines to harness the immune system against tumors, even in cases where traditional measures of success, such as tumor shrinkage, are not observed [80]. Beyond sipuleucel-T, other cancer vaccines have been explored in clinical trials. For instance, talimogene laherparepvec (T-VEC), an oncolytic virus-based vaccine approved for melanoma, has shown promise in early-stage melanoma in the OPTiM trial [47]. In contrast, the MAGE-A3 vaccine, tested in the MAGRIT trial, did not show a survival benefit in either NSCLC or melanoma [48]. Meanwhile, the GVAX pancreatic cancer vaccine, combined with the checkpoint inhibitor CRS-207, has demonstrated some survival improvement in pancreatic cancer patients in Phase II trials [49]. Similarly, PROSTVAC, a viral vector vaccine targeting prostate-specific antigen (PSA), did not meet its primary survival endpoint in Phase III trials but is being studied further in combination therapies [81]. While some vaccines like sipuleucel-T and T-VEC have achieved clinical approval, others face ongoing challenges, although research into cancer vaccines remains promising (Table 2).

### 5.4. Combination Therapy Trials (Combination of Atezolizumab and Nab-Paclitaxel)

The IMpower150 trial was a key Phase 3 study that assessed the efficacy of combining atezolizumab (a PD-L1 inhibitor) with nab-paclitaxel and chemotherapy in patients with advanced non-squamous NSCLC [82]. Atezolizumab works by blocking the PD-L1 protein on tumor cells, which typically binds to the PD-1 receptor on T cells, suppressing the immune response. By inhibiting this interaction, atezolizumab reactivates T cells to recognize and attack cancer cells. Nab-paclitaxel, a nanoparticle albumin-bound form of paclitaxel, enhances drug delivery to tumors by targeting albumin receptors overexpressed on cancer cells, improving treatment precision and reducing side effects [83]. The trial enrolled over 1,200 patients with advanced or metastatic non-squamous NSCLC who had not received prior chemotherapy. The patients were divided into three groups: one received atezolizumab with carboplatin and paclitaxel, another received atezolizumab with bevacizumab (an anti-angiogenic agent) and chemotherapy, and the control group received bevacizumab with standard chemotherapy. Bevacizumab was added to improve immune access to tumors by reducing their blood supply. The results were remarkable; patients receiving the combination of atezolizumab, bevacizumab, and chemotherapy had a median overall survival (OS) of 19.2 months, compared to 14.4 months in the control group, and a median progression-free survival (PFS) of 8.3 months vs. 6.8 months in the control group [84]. Patients with high PD-L1 expression benefited most, but even those with low or negative PD-L1 expression showed improved outcomes, highlighting the broad potential of this treatment strategy. Side effects, including fatigue, nausea, hair loss, and hematologic toxicities (e.g., anemia, neutropenia), were generally manageable. Immune-related side effects, such as pneumonitis and colitis, were observed but could be effectively managed with immunosuppressive therapies. The IMpower150 trial demonstrated the value of combining immunotherapy with chemotherapy and anti-angiogenic agents, offering a more comprehensive and effective treatment for advanced NSCLC [85].

### 5.5. Translating Clinical Trial Success into Real-World Clinical Practice

While the efficacy of CAR-T therapies, ICIs, and cancer vaccines demonstrated in clinical trials is noteworthy, translating these successes into real-world clinical practice poses significant challenges. Key barriers include high costs, patient selection, and the necessary infrastructure.

(i) Cost: CAR-T therapies can exceed USD 400,000 per infusion, while ICIs and combination regimens also incur substantial costs. These financial burdens hinder broad adoption, particularly in resource-limited areas, raising concerns about reimbursement and financial toxicity for patients and healthcare systems.
(ii) Patient Selection: The effectiveness of immunotherapies relies on accurately identifying patients most likely to benefit from the treatment. For CAR-T therapy, this involves assessing tumor burden and performance status, while ICIs utilize biomarkers like PD-L1 expression and tumor mutational burden (TMB). Ongoing research aims to develop more robust biomarkers for better patient stratification. Clinical trials for combination therapies emphasize the need for careful selection to maximize efficacy.
(iii) Real-World Data and Practice Guidelines: Clinical trial outcomes have shaped treatment guidelines, recommending CAR-T therapies for relapsed/refractory large B-cell lymphoma and ICIs as the standard for advanced NSCLC, melanoma, and renal cell carcinoma. However, real-world results may differ due to patient variability and healthcare infrastructure differences, making real-world data essential for assessing the therapies’ broader applicability. Cancer vaccine trials also contribute to evolving guidelines and treatment paradigms.
(iv) Infrastructure and Expertise: Administering CAR-T therapy and ICIs requires specialized centers with trained staff capable of managing complex toxicities like cytokine release syndrome (CRS) and immune-related adverse events (irAEs). The shift toward multidisciplinary care teams has redefined cancer treatment delivery, ensuring comprehensive management of these innovative therapies.

## 6. Personalized Immunotherapy

Personalized immunotherapy is revolutionizing cancer treatment by utilizing precision medicine to create tailored therapies that align with the unique characteristics of each patient’s tumor. This innovative approach aims to maximize treatment effectiveness while minimizing side effects by focusing on the distinct molecular and genetic makeup of the tumor, as well as the patient’s overall health and genetic predispositions. In precision medicine for tumor immunotherapy, extensive genomic, proteomic, and transcriptomic analyses guide treatment choices. By pinpointing specific genetic mutations, changes in signaling pathways, and immune system profiles, this approach enables the development of targeted therapies that are more likely to succeed for individual patients. As a result, treatments are not only more effective but also less likely to induce unnecessary side effects, as they specifically exploit the tumor’s vulnerabilities rather than apply a generic solution [86].

The integration of AI and machine learning is further enhancing personalized immunotherapy, particularly in predicting patient responses to next-generation immunotherapies. These technologies enable the analysis of vast datasets, allowing for the identification of patterns that predict patient responses to various treatments. For example, the IBM Watson for Oncology platform utilizes natural language processing and machine learning to analyze clinical data and scientific literature, helping oncologists predict the effectiveness of next-generation therapies such as CAR-T cell therapy and ICIs. Additionally, Tempus Labs has developed algorithms that integrate genomic sequencing data with clinical outcomes, enabling oncologists to identify the most promising treatment options based on a patient’s genetic mutations, particularly in the context of novel immunotherapeutic agents. Another promising example is the D-CRAFT (data-driven clinical recommendations for personalized therapy) algorithm, currently in development, which leverages machine learning to analyze large-scale patient data, including demographics, treatment history, and genomic profiles, to offer tailored therapy recommendations for advanced immunotherapies [87].

Biomarker-based immunotherapy is a cornerstone of this personalized strategy. Biomarkers, such as certain proteins, gene expressions, or genetic mutations, provide crucial insights into the tumor’s behavior and its interaction with the immune system. For instance, levels of PD-L1 expression can help predict how well checkpoint inhibitors will work, enabling clinicians to choose the most effective immunotherapy for each patient. Similarly, tumor mutational burden can identify patients more likely to respond positively to specific immunotherapies, further refining treatment decisions [88]. Addressing tumor heterogeneity is essential in developing personalized treatment plans, as tumors often display significant variability both within their structure and between different tumors in the same patient. This complexity requires personalized treatment strategies that incorporate comprehensive profiling techniques to understand the diverse mutations and immune evasion mechanisms present in the tumor [89,90]. Such insights allow for the creation of multifaceted treatment regimens that target various aspects of the tumor’s biology and adapt as the tumor evolves. In summary, personalized immunotherapy blends precision medicine, biomarker-driven approaches, and strategies to tackle tumor heterogeneity, resulting in a customized treatment strategy that enhances efficacy while reducing toxicity. This approach not only increases the likelihood of successful outcomes but also propels the field of oncology forward by offering more precise and individualized patient care.

## 7. Challenges and Limitations of Tumor Immunotherapy

While tumor immunotherapy holds immense promise for transforming cancer treatment, several challenges and limitations must be addressed to improve its effectiveness and accessibility. One major obstacle is immunotherapy resistance, with many patients experiencing minimal or no benefit from these therapies. Resistance can develop through various mechanisms, such as tumor-induced immune suppression, genetic mutations that alter how antigens are presented, or the upregulation of alternative immune checkpoints. To combat this resistance, researchers are actively exploring strategies like combination therapies that target multiple pathways simultaneously, novel agents that bypass known resistance mechanisms, and personalized treatment approaches that adapt to the evolving profiles of tumors and immune responses [91].

Managing adverse effects presents another critical challenge in immunotherapy. While these treatments can provide significant benefits, they may also lead to toxicity and autoimmune reactions. Immune-related adverse events (irAEs) can include inflammation of healthy tissues, such as colitis, dermatitis, or pneumonitis, stemming from the enhanced immune activity triggered by the therapy. Autoimmune reactions, in which the immune system mistakenly attacks the body’s own cells, also pose risks. Researchers are developing safer dosing regimens, such as fractional dosing or intermittent therapy schedules, to minimize the incidence and severity of irAEs. Furthermore, investigational agents such as immune modulators and targeted therapies are being studied to selectively mitigate these adverse effects without compromising the anti-tumor efficacy of the primary treatment. Effective management of irAEs necessitates early detection and intervention, with established protocols to address these events and mitigate their impact on a patient’s overall health. This may involve the use of immunosuppressive medications, therapy adjustments, or supportive care measures [92].

Cost and accessibility are significant barriers to the widespread adoption of next-generation immunotherapies. The development, production, and administration of these advanced treatments often come with high costs, making them less accessible to many patients. The complexity of manufacturing these treatments, particularly for personalized therapies like CAR-T cell treatments, further exacerbates this issue. Efforts to tackle these challenges include reducing production costs through advances in manufacturing technologies, enhancing treatment delivery efficiency via streamlined protocols, and expanding access through policy changes, improved insurance coverage, and initiatives aimed at making these therapies more affordable and available in various healthcare settings. Additionally, collaborative efforts among academic institutions, industry partners, and regulatory bodies are being pursued to facilitate the development of standardized guidelines for broader implementation of these therapies.

In summary, addressing the challenges of immunotherapy resistance, managing adverse effects, and tackling cost and accessibility issues are essential for unlocking the full potential of these therapies [93]. Ongoing research, innovative strategies, and systemic improvements are vital to maximizing the benefits of tumor immunotherapy and ensuring that its advancements are effectively and equitably applied to diverse patient populations.

## 8. Future Directions in Tumor Immunotherapy

The future of tumor immunotherapy is poised for transformative advancements, driven by breakthroughs in biomaterials, nanotechnology, and AI. These innovations promise to enhance the precision, efficacy, and accessibility of immunotherapy, creating new frontiers in cancer treatment.

### 8.1. Advancements in Biomaterials

Biomaterials are playing an increasingly critical role in improving the precision and effectiveness of immunotherapy. Next-generation biomaterials are being engineered to create sophisticated drug delivery systems that allow for more accurate targeting of tumor sites, while controlling the release of therapeutic agents in response to environmental cues. For example, biodegradable polymers and hydrogels are being designed to respond to changes in the TME, such as pH or temperature, enabling the more precise delivery of immunotherapies [94]. Engineered nanoparticles and microparticles are being tailored to encapsulate immunotherapeutic agents, reducing systemic toxicity by delivering drugs directly to the tumor site, thereby improving therapeutic outcomes. A recent study demonstrated that nanoparticle-based biomaterial scaffolds achieved a 50% improvement in drug retention at tumor sites compared to the results for traditional delivery systems, significantly reducing off-target effects [95].

Additionally, biomaterial-based scaffolds and implants are being explored for localized, sustained release of immuno-modulatory agents. These materials can act as platforms for long-term immune activation, where immunotherapies are delivered continuously to the tumor environment, enhancing immune system engagement while minimizing systemic exposure. For instance, hydrogel-based implants designed for the localized delivery of interleukin-2 have shown promising results in boosting immune responses, with fewer side effects than those for systemic administration [94]. Such advancements are key to overcoming current limitations in immunotherapy delivery and enhancing both treatment efficacy and patient safety.

### 8.2. Nanotechnology for Precision and Reduced Toxicity

Nanotechnology is reshaping the landscape of immunotherapy through novel methods for drug delivery, imaging, and treatment monitoring. The small size and unique properties of nanoparticles enable them to be precisely engineered for the targeted delivery of immunotherapeutic agents, reducing off-target effects and improving treatment specificity [95]. For example, lipid nanoparticles and gold nanostructures are being designed to deliver checkpoint inhibitors or CAR-T cells directly to the tumor, reducing the risk of damage to healthy tissues. A recent clinical trial using nanoparticle-mediated delivery of checkpoint inhibitors reduced immune-related adverse events (irAEs) by nearly 40% compared to the results for traditional administration routes [96]. The integration of nanotechnology is expected to not only improve the precision of immunotherapy but also to reduce treatment toxicity and improve patient outcomes. Ongoing developments in targeted nanoparticle systems have already shown substantial reductions in systemic side effects, making these therapies more tolerable for patients, especially in combination regimens.

### 8.3. Artificial Intelligence (AI)-Driven Personalization and Decision Making

AI is emerging as a game-changer in tumor immunotherapy, driving advancements in patient selection, treatment optimization, and clinical decision making. AI and machine learning algorithms are being employed to analyze vast datasets, including genomic, proteomic, and clinical information, enabling the identification of biomarkers that predict patient responses to immunotherapies [97]. These AI-driven tools can detect patterns in patient data that are often imperceptible via traditional analyses, allowing for the creation of personalized treatment plans tailored to individual patient profiles. For instance, companies like Tempus and Foundation Medicine are utilizing AI to analyze genetic and molecular data from cancer patients to predict how these patients will respond to immunotherapies like checkpoint inhibitors and CAR-T cell therapies. AI has also been used in projects such as IBM Watson for Oncology, which integrates patient data to recommend immunotherapies based on individual tumor characteristics, improving the accuracy of treatment selection by 30%. By incorporating AI into clinical workflows, clinicians can better identify which patients are most likely to benefit from specific therapies, improving response rates and reducing unnecessary treatments. In clinical trial design, AI-powered platforms like Path-AI are being used to analyze pathology slides and genetic data to uncover predictive markers for immunotherapy efficacy, accelerating drug development timelines and improving the efficiency of clinical trials. This integration of AI into both clinical practice and research is expected to streamline the development of new therapies, shorten the time to market for innovative treatments, and enhance patient outcomes by providing more precise and informed treatment strategies [97,98].

## 9. Conclusions

Tumor immunotherapy represents one of the most promising frontiers in cancer treatment, offering the potential for long-term remission and even cures for some patients. However, several challenges remain that require immediate and focused attention. Overcoming immunotherapy resistance, which continues to limit the success of these therapies for a significant number of patients, is a critical priority. Future research should intensify efforts to unravel the mechanisms of resistance and develop strategies that can overcome these barriers, whether through combination therapies, new immune checkpoint inhibitors, or personalized immunotherapies that adapt to the evolving nature of tumors. Another urgent area for future research is the reduction of the costs associated with next-generation immunotherapies. The high financial burden of these treatments restricts their accessibility, particularly in low-resource settings. There is an essential need for innovative approaches that can lower production costs, streamline treatment delivery, and expand access through improved healthcare infrastructure and policy changes. Additionally, more work is needed to refine the management of immune-related adverse events (irAEs) [99]. Developing safer dosing regimens, early detection systems, and personalized protocols will be essential to minimizing the risks of toxicity and autoimmune reactions, while maintaining treatment efficacy.

In conclusion, while tumor immunotherapy has advanced significantly, immediate progress is needed in addressing resistance, reducing costs, and improving patient safety. Collaborative efforts between researchers, clinicians, and policymakers will be essential to overcoming these hurdles and fully realizing the transformative potential of immunotherapy for a broader range of patients. Only through continued innovation and systemic improvements can we ensure that the benefits of these therapies are both effective and accessible to all who need them [100].

## 10. Clinical Outcomes

(i) Next-generation therapies, including ICIs, CAR-T cell therapy, and cancer vaccines, have demonstrated notable improvements in patient survival, particularly in traditionally hard-to-treat cancers such as melanoma, NSCLC, and hematologic malignancies.
(ii) Innovative combination therapies and personalized, biomarker-driven treatments have enhanced immunotherapy’s effectiveness, extending its reach to more cancer types, including those once resistant to treatment.

## Figures and Tables

**Table 1:** Comprehensive overview of key immunotherapeutic modalities in cancer treatment, with relevant references for enhanced insight into mechanisms and clinical applications.

Therapy Type	Target Tumor Type	Description	Role in Cancer Treatment	Key Strategies and Approaches	Challenges and Considerations	References
CAR-T Cell Therapy	Leukemia, lymphoma, trials in solid tumors	Genetically engineered T cells designed to target specific cancer antigens.	Highly effective in hematologic malignancies; potential in solid tumors.	Use of engineered T cells recognizing cancer antigens.	Severe side effects (e.g., cytokine release syndrome); high costs.	[9,16,23]
Checkpoint Therapy	Melanoma, NSCLC, RCC	Drugs blocking inhibitory pathways (e.g., PD-1, CTLA-4) in immune cells.	Effective in melanoma, lung, and other solid cancers.	Immune checkpoint inhibitors, often combined with other therapies.	Resistance development; autoimmune side effects; limited efficacy.	[3,6,13]
**Vaccines**	Prostate, melanoma, cervical cancer	Vaccines designed to elicit immune responses against cancer-specific antigens.	Aim to prevent cancer development or recurrence.	Prophylactic and therapeutic vaccines; personalized cancer vaccines.	Limited efficacy; identifying optimal antigens.	[18,24,25]
**Antibodies**	Breast cancer, lymphoma, leukemia	Monoclonal antibodies targeting specific cancer antigens.	Direct cancer cell killing or immune system activation.	Anti-CD20 (Rituximab), HER2-targeted therapies (Trastuzumab).	Resistance development; infusion reactions.	[26,27]
**Combination Therapy**	Various cancers	Combining multiple modalities (e.g., immunotherapy + chemotherapy).	Aims to improve survival and address resistance.	Biomarker-driven personalized combinations.	Potential for increased toxicity and complex dosing strategies.	[28,29,30]

**Table 2:** Summary of prominent immunotherapies: Insights into TIM-3, LAG-3, TIGIT, Yescarta, and Kymriah regarding tumor targeting, efficacy, and toxicity profiles, alongside T-VEC, MAGE-A3, and GVAX.

Therapy	Target (Tumor Types)	Mechanism of Action	Efficacy	Toxicity	References
**TIM-3 Inhibitors**	Multiple cancer types (NSCLC, melanoma, solid tumors)	Blocks the TIM-3 pathway, enhancing T cell activation.	Shows promise in improving T cell responses in combination with PD-1 inhibitors. Clinical trials are ongoing.	Immune-related adverse events (irAEs) including colitis, pneumonitis, liver inflammation, dermatitis.	[36,39]
**LAG-3 Inhibitors**	Melanoma, NSCLC, RCC	Inhibits the LAG-3 receptor, promoting T cell activity.	LAG-3 inhibitors like relatlimab have shown efficacy in combination with PD-1 inhibitors. Improved response rates.	irAEs similar to PD-1 inhibitors, but generally manageable. Fatigue, rash, diarrhea.	[40,41]
**TIGIT Inhibitors**	NSCLC, SCLC, solid tumors	Blocks the TIGIT pathway, enhancing immune response.	TIGIT inhibitors have shown modest responses in clinical trials, often used in combination with PD-1 inhibitors.	Mild to moderate irAEs, including rash, fatigue, pneumonitis, and colitis.	[41,42]
**Yescarta (Axicabtagene ciloleucel)**	Large B-cell lymphoma	CAR-T therapy targeting CD19 antigen on B cells.	High efficacy in relapsed/refractory B-cell lymphoma. Achieved ~51% complete remission rate in clinical trials.	Cytokine release syndrome (CRS), neurotoxicity (ICANS), fever, fatigue, hypotension.	[43,44]
**Kymriah (Tisagenlecleucel)**	B-cell ALL, DLBCL	CAR-T therapy targeting CD19 antigen on B cells.	Achieved 68% overall response rate in pediatric B-cell ALL. Long-term remissions observed.	CRS, neurotoxicity, fever, neutropenia, thrombocytopenia, infections.	[45,46]
**Talimogene laherparepvec (T-VEC)**	Melanoma (advanced stage)	Oncolytic virus that selectively infects and destroys cancer cells.	Demonstrated modest efficacy in melanoma. Achieved 26.4% overall response rate in Phase III trials.	Mild toxicity: fever, fatigue, injection site reactions. Mild irAEs like rash or flu-like symptoms.	[47]
**MAGE-A3 Vaccine**	NSCLC, melanoma	Targets MAGE-A3 antigen to stimulate an immune response.	Failed to show significant efficacy in multiple Phase III trials for both NSCLC and melanoma.	Minimal toxicity, mostly mild irAEs, including fatigue and injection site reactions.	[48]
**GVAX**	Pancreatic cancer, prostate cancer	Whole-cell vaccine that stimulates the immune system against tumor antigens.	Limited efficacy. Initial trials in prostate cancer showed no significant survival benefit.	Generally mild toxicity, fever, fatigue, injection site reactions.	[49]

## Data Availability

No new data were created or analyzed in this study. Data sharing is not applicable to this article.

## Abbreviations

AI—artificial intelligence; APC—antigen-presenting cell; CAR-T—chimeric antigen receptor T cell; CTLA-4—cytotoxic T-lymphocyte-associated protein 4; DC—dendritic cell; FDA—Food and Drug Administration; ICIs—immune checkpoint inhibitors; IL—interleukin; MDSC—myeloid-derived suppressor cells; NK—natural killer (cells); NSCLC—non-small cell lung cancer; PD1—programmed cell death protein 1; PDL1—programmed death-ligand 1; RCC—renal cell carcinoma; TCR—T cell receptor; TIL—tumor-infiltrating lymphocytes; TME—tumor microenvironment; Treg—regulatory T cells; VEGF—vascular endothelial growth factor.
